# Altered Composition of Liver Proteasome Assemblies Contributes to Enhanced Proteasome Activity in the Exceptionally Long-Lived Naked Mole-Rat

**DOI:** 10.1371/journal.pone.0035890

**Published:** 2012-05-02

**Authors:** Karl A. Rodriguez, Yael H. Edrey, Pawel Osmulski, Maria Gaczynska, Rochelle Buffenstein

**Affiliations:** 1 Sam and Ann Barshop Institute for Aging and Longevity Studies, University of Texas Health Science Center at San Antonio, San Antonio, Texas, United States of America; 2 Department of Physiology, University of Texas Health Science Center at San Antonio, San Antonio, Texas, United States of America; 3 Department of Molecular Medicine, University of Texas Health Science Center at San Antonio, San Antonio, Texas, United States of America; 4 Department of Cellular and Structural Biology, University of Texas Health Science Center at San Antonio, San Antonio, Texas, United States of America; University of Pittsburgh, United States of America

## Abstract

The longest-lived rodent, the naked mole-rat (Bathyergidae; *Heterocephalus glaber*), maintains robust health for at least 75% of its 32 year lifespan, suggesting that the decline in genomic integrity or protein homeostasis routinely observed during aging, is either attenuated or delayed in this extraordinarily long-lived species. The ubiquitin proteasome system (UPS) plays an integral role in protein homeostasis by degrading oxidatively-damaged and misfolded proteins. In this study, we examined proteasome activity in naked mole-rats and mice in whole liver lysates as well as three subcellular fractions to probe the mechanisms behind the apparently enhanced effectiveness of UPS. We found that when compared with mouse samples, naked mole-rats had significantly higher chymotrypsin-like (ChT-L) activity and a two-fold increase in trypsin-like (T-L) in both whole lysates as well as cytosolic fractions. Native gel electrophoresis of the whole tissue lysates showed that the 20S proteasome was more active in the longer-lived species and that 26S proteasome was both more active and more populous. Western blot analyses revealed that both 19S subunits and immunoproteasome catalytic subunits are present in greater amounts in the naked mole-rat suggesting that the observed higher specific activity may be due to the greater proportion of immunoproteasomes in livers of healthy young adults. It thus appears that proteasomes in this species are primed for the efficient removal of stress-damaged proteins. Further characterization of the naked mole-rat proteasome and its regulation could lead to important insights on how the cells in these animals handle increased stress and protein damage to maintain a longer health in their tissues and ultimately a longer life.

## Introduction

The naked mole-rat, *Heterocephalus glaber*, is the longest-lived rodent on record, with a maximum lifespan greater than 30 years [1]. Not only does this animal live 8 to 10 times longer than similar-sized mice but this species also shows prolonged maintenance of cancer-free good health and reproductive potential well into its third decade [2]. In contrast, mice live half as long as predicted on the basis of body size [3] and start showing age-related changes after only one year of life [4]. The recently completed naked mole-rat genome [5] has revealed that several gene families associated with protein degradation are expanded. Furthermore, RNA sequence analysis has also shown that many genes associated with protein homeostasis including chaperones, ubiquitin conjugating enzymes and proteasome subunits are overexpressed relative to mice [5], [6]. Young naked mole-rats (2 year old) show 2-8-fold higher levels of oxidative damage to proteins, lipids and DNA than physiologically age-matched mice (4 months) and similar levels of damage to that observed in chronologically age-matched (2-yr old) mice [7]. Despite high levels of oxidative damage even at a young age, ubiquitinylated proteins are maintained at lower levels than mice at both young and old ages [8], suggestive of less accumulation of damaged or misfolded proteins. Collectively, these findings may be indicative of a highly efficient ubiquitin-proteasome system (UPS) in the naked mole-rat.

The UPS is responsible for the regulated degradation of proteins [9]. As such, the proteasome is generally regarded as an integral component in the maintenance of protein quality control. This in turn may play a critical role in the maintenance of healthspan and longevity. The UPS is complex, highly specific and tightly regulated involving several hundred specific proteins. Proteasomes recognize ubiquitinylated substrates, and cleave the polypeptide substrate into smaller peptides for the overall purpose of maintaining cell homeostasis [10], [11]. The substrates degraded fall into two classes: a) short-lived proteins usually involved in cell-cycle control, growth or transcription which are mostly found in the cytosol and nucleus and b) misfolded or damaged proteins [12], [13]. Removal of long-lived, damaged proteins occurs in the cytosol, while quality control of newly synthesized proteins takes place near the endoplasmic reticulum. The subcellular distribution of the proteasome varies depending on the tissue and stage of the cell cycle, but in general 60 to 90% of active intact proteasomes reside in the cytosol. This subcellular fraction includes roughly 50% of proteasomes that interact with the cytosolic side of the endoplasmic reticulum and co-purify with the microsomes during differential centrifugation. [14]–[16]. The remaining 10% to 40% localize in the nucleus [14], [15] where proteasomes are responsible for the turnover of transcription factors, proteins involved in DNA repair, sister chromatid exchange, and DNA-damage checkpoint control [11], [17]–[19].

The proteasome consists of a core particle and one or two regulatory particles. In eukaryotes, the 20S core particle is composed of 28 subunits arranged into a cylinder built from four stacked heteroheptameric rings. The core has a molecular mass of approximately 700 kDa. The two internal rings contain three pairs of catalytic subunits (β1, β2, β5) that form six N-terminal threonine-based catalytic centers. The two outer rings consist of the alpha subunits and form gated channels leading into the catalytic chamber [20]–[22]. The core particle degrades polypeptides and unfolded proteins by cleaving peptide bonds on the carboxyl side of hydrophobic, basic or acidic residues. Action at these specific loci are referred to as “chymotrypsin-like” (ChT-L), “trypsin-like” (T-L) and peptidylglutamyl peptide hydrolyzing activity (also known as “caspase-like”/PGPH) and are carried out at the β5, β2, and β1 subunits respectively [23]. The 19S regulatory complex attaches to the 20S core on one or both sides. This “cap” regulates substrate uptake into the proteasome and is also responsible for deubiquitinylation allowing the substrate to unfold and spool through the catalytic core [9], [11]. The double-capped core is known as the 26S proteasome and is primarily responsible for proteolytic activity in a healthy cell. The 20S core without the regulatory particle is usually latent in cells because the N-termini of several α-subunits form a gate that most of the time blocks the entrance of substrates into the proteolytic chamber. Also, the core particle cannot recognize ubiquitin modifications or unfold protein substrates for degradation [24], [25]. However, *in vivo* the 20S core can degrade untagged proteins that are structurally unstable. Moreover *in vitro*, the 20S proteasome can degrade synthetic substrates in an ubiquitin-independent manner especially when the substrates are oxidatively damaged [26]–[28]. Recently, nuclear factor erythroid-derived 2 (Nrf2), a transcription factor controlling the cytoprotective signaling pathway, has been shown to upregulate proteasome activity and content in response to oxidative or electrophilic stress and may be responsible for both constitutive and stress induced transcription of proteasome genes [29].

Vertebrates also possess modified proteasomes in which the “housekeeping” beta catalytic subunits are replaced by structurally similar but not identical subunits (i.e. β1 to β1i/LMP2, β2 to β2i/MECL1 and β5 to β5i/LMP7). Both β1i and β5i are encoded within the major histocompatibility complex (MHC) class II region linking their function to antigen presentation and giving rise to their designation as immunoproteasomes [30], [31]. Immunoproteasomes are commonly produced in the spleen and thymus, but may also be found in lower levels in non-immunogenic tissues e.g. retina or liver [16], [32]. Canonically, the immunoproteasome regulates peptidolytic activity that results in a more efficient turnover of many MHC class I epitopes and other stress proteins [33]. Another multi-protein complex, proteasome activator 28 (PA28) can interact with the 20S core particle, forming 20S-PA28 or a hybrid proteasome (19S-20S-PA28) [34]. This PA28 is thought to be involved in the regulation of the immunoproteasome and upregulation of PA28 subunits reportedly protects against oxidative stress [35], [36]. Interestingly, higher levels of PA28 were reported in livers of young mice than in old mice [16]. Furthermore, with an experimentally induced immunoproteasome deficiency (i.e. either RNAi depletion or knockout animal studies) proteasomes present in the mouse retina could not respond to stress and were more susceptible to “oxidation- induced cell death” suggesting an oxidative, stress-related, non-immunogenic function for the immunoproteasome [37].

Given that naked mole-rats have higher levels of oxidative stress than mice [7] yet retain a long healthspan, in this study we evaluate possible mechanisms by which this is achieved. We question whether naked mole-rats have more proteasomes, rely on different catalytic subunits or subassemblies (i.e. immunoproteasomes) or if their proteasomes are more efficient and thereby maintain better protein quality control. We measured peptidolytic activity of ChT-L, T-L and PGPH peptidase activities of the proteasomes using model substrates in a commonly studied and well characterized tissue, the liver. This tissue was specifically chosen for several reasons. Liver is metabolically very active and rich in proteasomes. Proteasome activity in this organ has been well characterized in mice [16], [38], [39]. Moreover naked mole-rats show high levels of oxidative damage in liver samples [7], [8]. Using whole liver lysates as well as three subcellular fractions of these lysates, we test the hypothesis that naked mole-rats exhibit better quality control of both newly synthesized and/or longer-lived proteins.

## Results

### Naked mole-rats have higher proteasome activity than do mice

**Figure 1:**
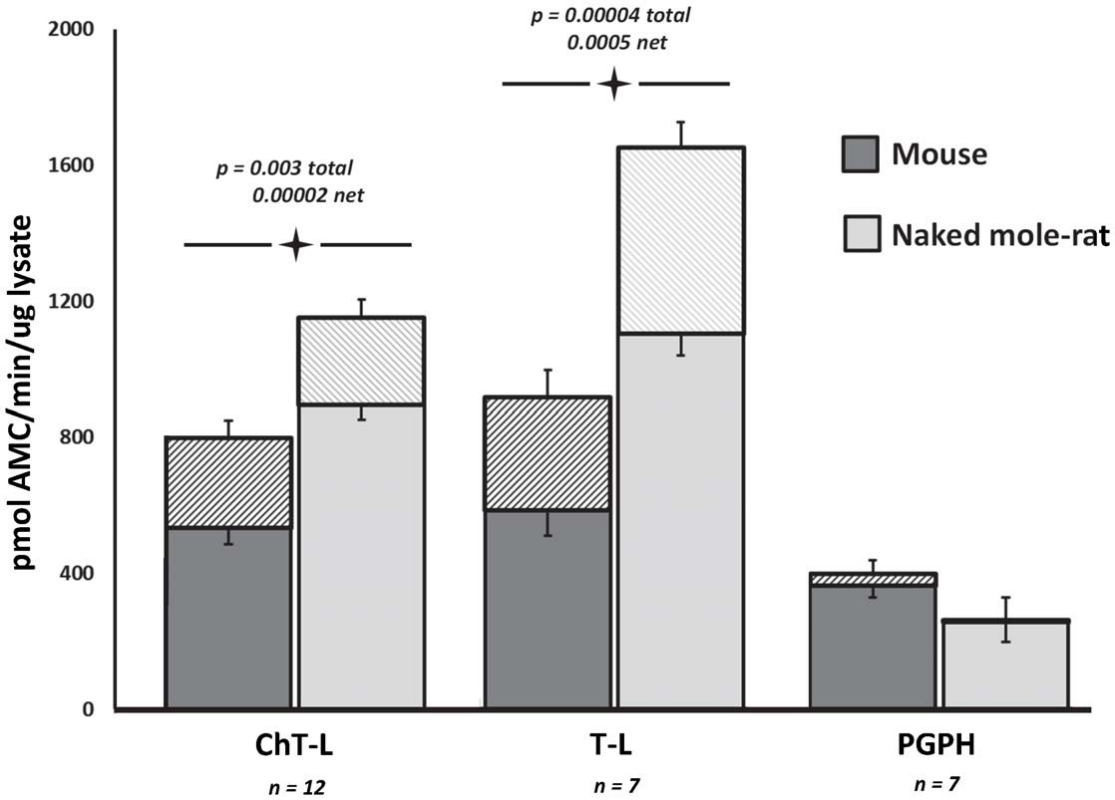
Chymotrypsin- and trypsin-like activities, but not the caspase-like activity were higher in the whole cell lysates from naked mole-rat than in mouse lysates. In each assay 50 µg of whole cell liver lysates from physiologically age-matched young mice (4 mo) and naked mole rats (2 yr) were used. The samples were incubated with 100 µM of substrate specific for the type of active center of the proteasome being measured. A saturating concentration of proteasome inhibitor N-(benzyl-oxycarbonyl) leucinyl-leucinal (MG132), determined by titration, was added to parallel samples. The difference of the fluorescence released with and without inhibitor was used as a measure of the specific peptidolytic activity of proteasome. Hatched lines indicate the amount of non-specific protease activity in excess of net specific proteasome activity. Values are means ± SE. Significant p-values are indicated in the figure.

Peptidolytic activity of the proteasome was measured in mouse and naked mole-rat whole liver tissue lysates using model peptide substrates specific for each of the three catalytic sites in the proteasome [16], [40], [41]. Since the peptides can be cleaved by other proteases that exhibit chymotrypsin-, trypsin- and caspase-like activity in the cells of these tissues, activity was assessed with and without the proteasome inhibitor N-(benzyloxycarbonyl) leucinyl-leucinylleucinal (MG132). The difference between these two measurements was assumed to exclusively result from proteasome activity. Naked mole-rats had double the rate of activity per microgram protein for both ChT-L activity and T-L total proteolytic activity in whole liver lysates compared to that seen in mice (Figure 1). Similarly after inhibition with MG132 proteasome inhibitor, proteasome specific (net) ChT-L activity was 1.5 times greater in the naked mole-rat and T-L activity was still twice that observed in mice (Figure 1). Of the three proteasome activities monitored, PGPH activity was the lowest in both species. Furthermore, it showed no significant difference between species, nor was there any distinct change between the total and net activities (Figure 1).

### High naked mole-rat proteasome activity stems from an increased specific activity

**Figure 2:**
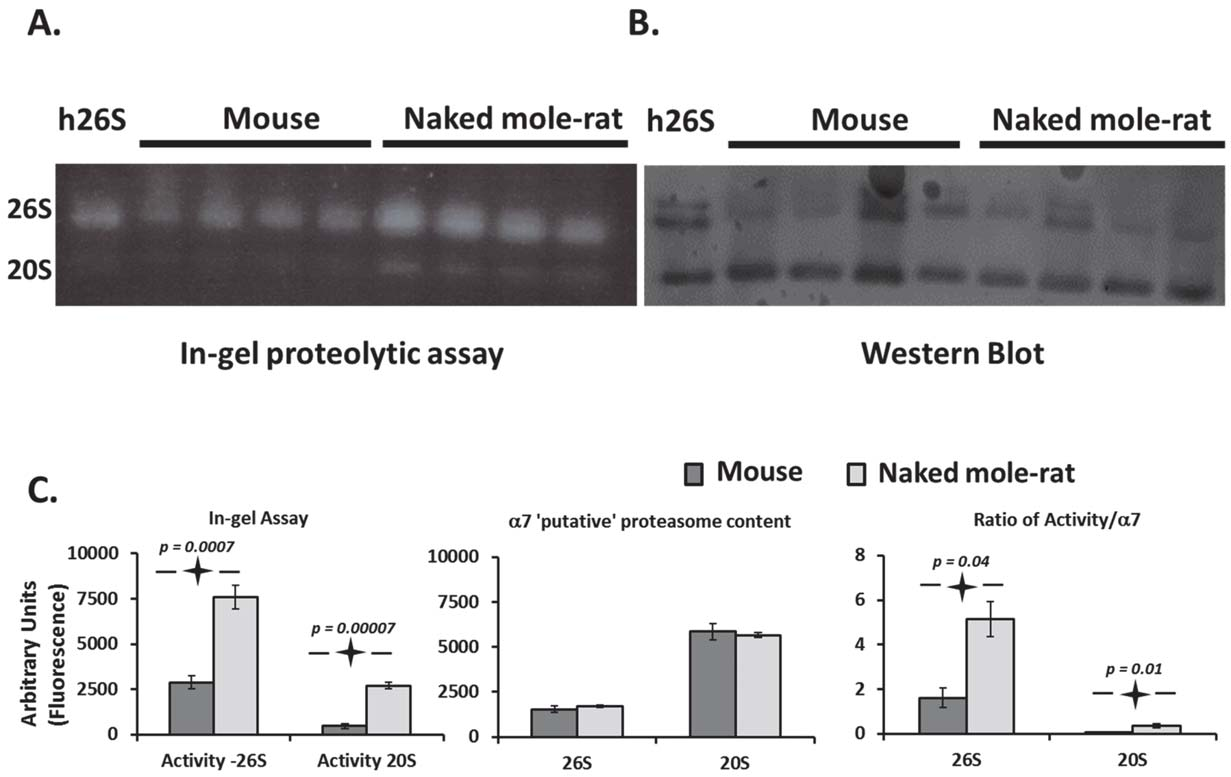
The higher proteasome activity observed in naked mole-rats when compared to mice stemmed from an increased specific activity of the 26S proteasomes. (A) A zymogram of the in-gel proteolytic assay revealed that naked mole-rat extracts exhibit higher 26S and 20S activity when compared to mice. (B) Immunoblot of the same native gel transferred to a PVDF membrane and probed with an antibody specific for the α7 proteasome subunit showed that the naked mole-rat had more 26S proteasome than mice yet both species had similar levels of 20S proteasome. (C) Quantitation of the native gel zymogram and Western blots. Both panels A and B are representative of several experiments the quantitation of which was conducted over 12 samples. The zymogram indicated that naked mole rats had about 3 times higher activity of 26S proteasome and 5 times higher activity for 20S proteasome subassemblies than mice. The content of α7 subunit in both the 26S band and 20S band was similar in both species. We also noticed that the content of α7 subunit in the 20S band was much higher than in 26S band in both species. However, the detected low activity in this band indicated that 26S complexes displayed more than 10 times higher specific activity than 20S complexes and specific activity in the naked mole-rat was 3 to 5 times higher for 26S and 20S activity. Histogram values shown are means ± SE. Significant p-values are indicated in the figures.

**Figure 3:**
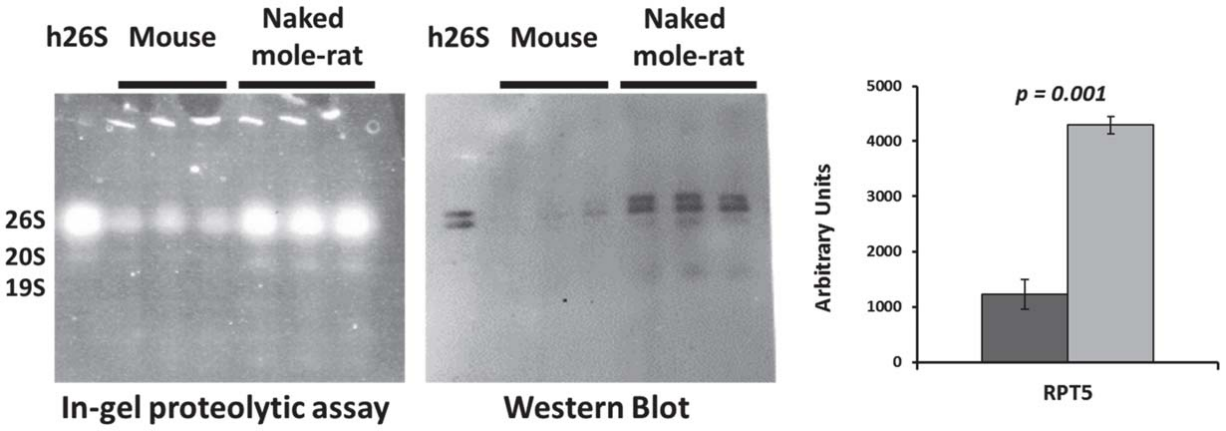
Native gel electrophoresis also showed higher levels of a key 19S subunit. We quantitated protein levels in immunoblots of liver tissue lysates probed with antibody against the 19S ATPase RPT5. Analysis revealed a three-fold higher (p<0.001) protein level in the naked mole-rats compared to mice. Samples from three different individuals of each species were used.

In order to confirm the observed higher activity of the proteasomes in the enzymatic assay and also evaluate species differences in the subassembly composition of proteasomes, ChT-L activity of lysates was also measured using non-denaturing polyacrylamide gels (‘native gels’). In-gel assays confirmed the high ChT-L activity in naked mole-rats and revealed that this corresponded to a significantly higher activity in the 26S assembly (Figure 2A, C left panel). The activity of 20S was also four times higher (p<0.001) in naked mole-rats, (Figure 2A, C left panel). Immunoblot analyses following transfer of proteins separated by native gel revealed that both naked mole-rats and mice had similar levels of α7 protein co-migrating with the activity represented by the 26S proteasome band (Figure 2B, C middle panel). The amounts of the α7 subunit in the region of 20S proteasome were also about the same in both species (Figure 2B, C middle panel). This constitutive proteasome subunit is considered a reasonable estimate of proteasome content [16] and suggests that both species have similar amounts of proteasomes. 19SATPase subunits (RPT5), “regulatory caps” are critical in recognizing polyubiquitinylated proteins [42], may also be used as a marker of 26 s proteasome content [43] and pronounced differences between the estimated proteasome content using α7 and RPT5 were evident (Figure 2, 3). Naked mole-rat samples had higher RPT5 than mice (Figure 3), suggesting the presence of more intact 26 s proteasome subassemblies in the longer lived species. The discrepancy between the α7 and the RPT5 levels as indicators of proteasome content may reflect the dynamic nature of the catalytic core relative to the regulatory caps. While antibodies may recognize the epitopes on the regulatory caps, in an intact 26S proteasome it is possible that some of the α7 subunits may be hidden and this yield an underestimate of the total proteasome content.

The ratio of activity to α7 subunit content showed that 26S proteasomes were at least 15 times more active than 20S proteasome in both naked mole-rats and mice. Further, this ratio shows that 26S proteasome-specific ChT-L activity is greater in the naked mole-rat than in mice (Figure 2, C right panel). While the content of 20S proteasome-related specific activity was also higher in naked mole-rats, the markedly higher 26S proteasome activity suggest that the catalytic core aside from its own modest enzymatic functions likely also serves as a pool to maintain adequate amounts of the catalytically more specialized 26S proteasomes.

### The contents of both immunoproteasome and 19S regulatory subunits are higher in naked mole-rats then in mice

**Figure 4:**
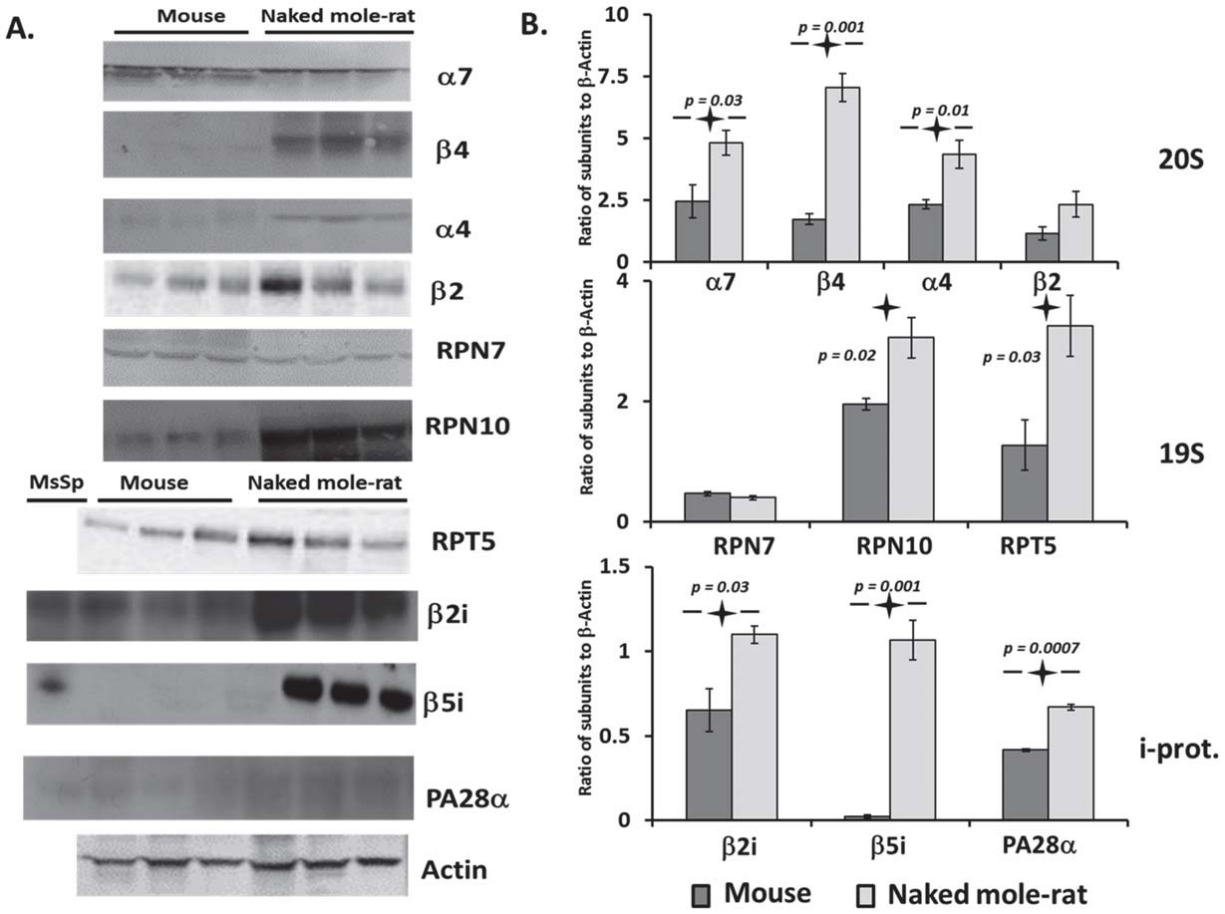
Analysis of proteasome subunit composition showed that naked mole-rats had higher protein content of 19S and immunoproteasome subunits than mice. (A) Representative Western blots with PVDF-transferred proteins were probed with antibodies specific for 20S, 26S and immunoproteasome subunits. Different content of various subunits revealed an upregulation of particular proteasome subassemblies. Samples from three different animals from each species were used per experiment and the experiment was repeated with samples from different animals at least one additional time to verify the outcome. The blots are representative of these sets. Actin was used as a loading control for our analyses. For immunoproteasome subunits, lysates from mouse spleen tissue (MsSp) were also used as a positive control. (B) Quantitation of Western blots grouped by 20S, 19S or immunoproteasome. Not only did naked mole-rats have higher content of constitutive non-catalytic subunits, but they also tended to have more immunoproteasome components (β2i, β5i, PA28α) than did mice. Naked mole-rats also had increased protein content of two critical 19S subunits (RPT5, RPN10). Values represent the mean ± SE with significant p-values highlighted in the figure.

There was no unincorporated α7 subunit detected in the naked mole-rat native gel immuno-blots (Figure 2B). However, we performed Western blot analysis under denaturing conditions (SDS-PAGE) using a panel of anti-proteasome subunits' antibodies. This revealed that both species had similar protein levels of the RPN7 and β2 proteasome subunits (Figure 4 A, B). Naked mole-rats, however, had higher levels of several other constitutive subunits, (namely α7, α4, and β4; Figure 4 A, B). Levels of α7 were two-fold higher in naked mole-rat lysates when compared to mice whereas α4 and β4 were three and four-fold higher in lysates from the longer lived species (Figure 4 A, B). These diverse trends in changes of the total content of constitutive and catalytic proteasome subunits that should exist in relatively equal amounts [9], [44] can be explained by upstream upregulation of the genes responsible for those protein products or by a mixed population of proteasome assemblies. To test the latter hypothesis, the contents of two immunoproteasome catalytic subunits β5i and β2i were also measured. Both these immunoproteasome subunits were present at higher amounts in the naked mole-rat lysates than in mouse tissues (Figure 4 A, B). This increase in the content of immunoproteasomes in liver lysates could explain the increase in constitutive subunits with no corresponding increase in housekeeping catalytic subunits. Similarly, the subunit of PA28α activator associated with the immunoproteasome showed increased protein levels in naked mole-rats when compared to mice. (Figure 4 A, B).

RPN10 protein is a key component of the proteasome 19S cap, stabilizing its structure and providing one of intrinsic ubiquitin receptors [45]. This protein level was higher in the naked mole-rat lysates compared to mice (Figure 4 A, B) suggesting higher levels of 26S proteasome. Both the high levels of RPN10 and RPT5 in naked mole-rat lysates than that in mice support the 26S findings from the native gel (Figure 3; Figure 4 A, B).

### The source of higher proteasome activity in naked mole-rats comes from the cytosolic fraction

**Figure 5:**
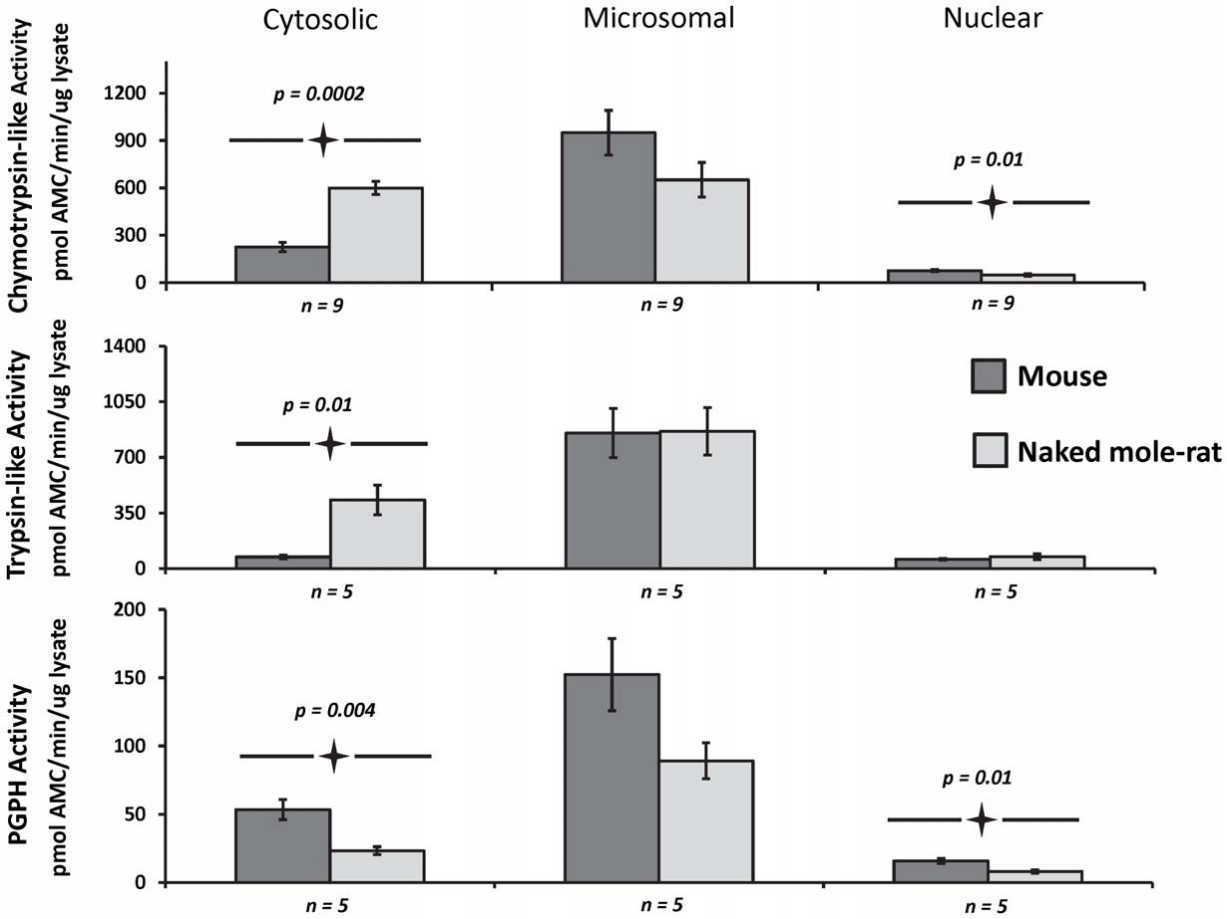
Specific peptidase activities in the cytosolic, microsomal, and nuclear fractions of naked mole-rat and mouse liver lysates showed pronounced species differences. Although the microsomal proteasome subfraction had the highest activity in both species and activity was similar in both species, marked differences were evident in the cytosolic fraction. Naked mole-rats had almost 3-fold higher ChT-L and 6-fold higher T-L activities in these cytosolic fractions. PGPH activity showed an opposite trend in specific activity to that observed for ChT-L and TL with mice having higher levels in the cytosolic fraction. Nuclear proteasomes showed significant differences in PGPH and ChT-L activity, but the activity was minimal compared to the activity in the other fractions. Specific activity was calculated as a reaction rate per amount of total protein and presented as the pmol of released AMC per 1 min per µg of total protein. A saturating concentration of proteasome inhibitor MG132, determined by titration was added to parallel samples. The difference of the fluorescence released with and without inhibitor was used as a measure of the peptidolytic activity of proteasome. Values shown represent the mean ± SE. Significant differences are highlighted in the figure along with their corresponding p-value.

**Figure 6:**
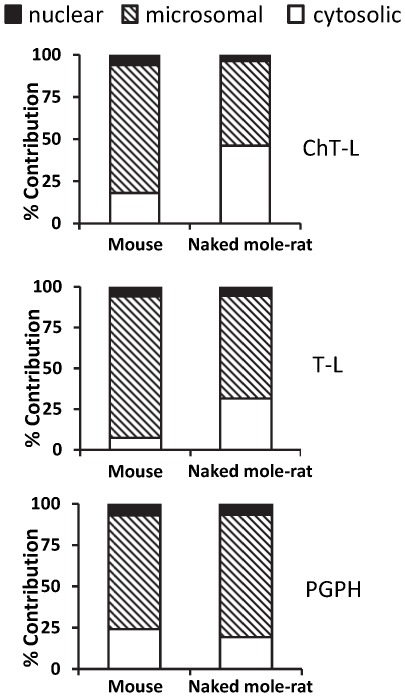
The largest species differences observed were the contribution of the cytosolic fraction for both ChT-L and T-L activities. Percent contribution of the total activity was calculated using the values of specific activities presented in Figure 4. In both species proteasome activity was highest in the microsomal fraction, but the microsomal contribution to the total activity within the lysate was greater in mouse samples (76%) than in naked mole-rat samples (50%) for ChT-L and this difference in % contribution was even greater for TL-activity (87%, 53% respectively). Nuclear fractions, regardless of the catalytic activity, only contributed 7% or less to the total activity in both species. PGPH activity showed a similar distribution within the subcellular fractions in both species. In sharp contrast the cytosolic fraction of ChT-L activity of naked mole-rats showed more than double (46%) the proportionate contribution to that of mice (18%) and this species difference was even greater for T-L activity (32% to 7%) in revealing that distributional differences in the observed total activity between species could be explained by interspecific differences in cytosolic activity.

In both species, the highest overall peptidolytic activities were observed in the microsomal fractions whereas the lowest overall activity was evident in the nucleus. The largest differences between species were observed in the cytosolic fraction (Figure 5). Both cytosolic ChT-L and T-L activity were 3× and 6× higher in naked mole-rat liver lysates than in mouse lysates, whereas PGPH activity was higher in mice than in naked mole-rats (Figure 5). This also amounted to a dramatic difference in the percent contribution of activity in the various fractions. For ChT-L the percentage contribution of the cytosolic fraction to overall activity was 46% and 17% in mole-rats and mice respectively, while for T-L activity the percentage contribution of the cytosolic fraction amounted to 32% for naked mole-rats versus 7% for mice (Figure 6). Naked mole-rat PGPH activity comprised less than five percent of the total activity in the cytosolic fraction, whereas in mouse lysates, it was closer to 25% (Figure 5, left panel; Figure 6). Nuclear proteasome activity also was low in comparison to the activities seen in the other fractions and in both species contributed about 10% of the total activities (Figure 6). Significantly higher nuclear ChT-L and PGPH activities were observed in mice; indeed in the nuclear fraction only T-L was higher in the naked mole-rat (Figure 5, right panels; Figure 6).

### The common transcription factors regulating immunoproteasome upregulation are increased in naked mole-rats when compared to mice

**Figure 7:**
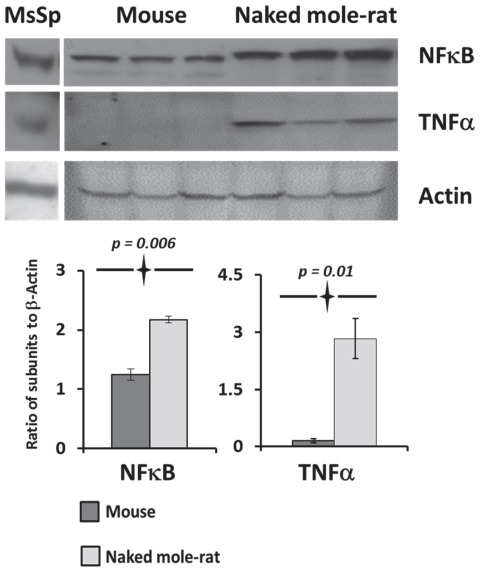
Levels of markers of an inflammatory response were higher in naked mole-rat than in mice. We quantitated protein levels in Western blot analyses of liver tissue lysates probed with anti NFκB and TNFα antibodies. Both NFκB and TNFα protein levels were more than two-fold higher (p≤0.01) in naked mole-rats. Samples from three different individuals of each species were used and the experiment was repeated with lysates from different animals several times to verify the outcome. The blots shown are representative of these experiments. Actin was used as a loading control and lysates from mouse spleen tissue (MsSp) represented a positive control for these immune-related markers.

Since high levels of immunoproteasome activity were evident in naked mole-rats liver lysates, markers for an inflammatory response (nuclear factor kappa-light-chain-enhancer of activated B cells (NFκB) and tumor necrosis factor-alpha (TNFα) were also measured. Both NFκB and TNFα had a higher protein expression in healthy naked mole-rat liver tissue than in mice as determined by Western blot analyses (Figure 7).

## Discussion

This study set out to test the hypothesis that long-lived naked mole-rats have more proteasome activity than do short-lived mice and revealed several key and/or surprising findings: 1) Compared to mice, naked mole-rats show similar amounts of proteasome (α7) content yet have higher rates of ChT-L and T-L activity cleaving both the hydrophobic and basic model substrates, respectively. In contrast, cleavage of the acidic substrate was similar in both species and appeared to play a minor role in protein degradation. 2) The higher proteasome activity observed in naked mole-rat tissue extracts over mouse tissue extracts was due to a higher 26S activity per unit of proteasome in the naked mole-rat. 3) The immunoproteasome was more active in liver tissue in healthy young naked mole-rats, compared to liver samples from healthy young mice. 4) The major contributor to this increased activity seen in naked mole-rats is the cytosolic immunoproteasome rather than its ‘housekeeping’ counterpart. Collectively, these data reveal that the naked mole-rat has highly efficient protein degrading machinery and thereby maintains high levels of protein quality control, constantly degrading misfolded and damaged proteins, thus maintaining steady state levels throughout life [8]. These actions contribute to the superior maintenance of protein integrity in the naked mole-rat. As such these may be key factors leading to their exceptional longevity and prolonged good health at ages equivalent to human nonagenerians, despite prior evidence of chronic oxidative stress evident even at very young ages [2].

These findings of enhanced protein degradation and concomitant protein turnover in an extraordinarily long-lived species concur with data based upon recent yeast mutant studies. Deletion of UBR2, an ubiquitin ligase that regulates Rpn4 turnover, results in elevated Rpn4 levels, which increases UPS capacity, significantly extending replicative aging and increasing resistance to proteotoxic stress [46]. Further, the yeast mutant cells with this higher proteasome capacity have higher protein turnover, especially of unstable and aggregation prone substrates [46]. Differential translation of UPS protein components is seen during oxidative or other cytotoxic stress in other species. In long-lived mutant worms, resistance to thermal stress and oxidative stress selectively increases protein translation [47]–[49]. In mammalian cells, oxidative stress signals an initial translation-independent activation of the UPS and increase in proteasome activity. This increased protein degradation in response to stress is then compensated by a subsequent and progressive increase in proteasome transcription and translation [28].

### Naked mole-rats show higher rates of post-hydrophobic peptide cleavage than mice

Hydrophobicity is one of the signals for selective degradation of hydroxyl radical modified proteins [50], and in naked mole-rats, increased proteasome activity is seen at the site responsible for cleavage after hydrophobic residues (ChT-L; Figure 1). When subjected to oxidative stress, neuronal cell crude lysates reportedly show an increase in ubiquitinylated and oxidized proteins and a concomitant increase in proteasome activity to eliminate damaged proteins [38]. Moreover, studies involving rat brain and liver preparations reveal if the degree of oxidative stress overwhelms the UPS system (e.g., following exposure to hydrogen peroxide (H_2_0_2_) and 4-hydroxynonenal), the increase in insoluble material may impair proteasome function and inhibit activity [51]. A recent study measuring proteasome activity in H_2_0_2_-treated mouse embryonic fibroblasts, reports that cells acclimate to the increased oxidatively-stressed environment and after an initial decline in proteasome activity, proteasome activity is increased [28]. The cells seemingly developed adaptability to the increased oxidatively-stressed environment. Similarly, we show that in healthy naked mole-rats, the increased proteasome activity is induced to most likely compensate for higher levels of oxidative stress and maintain protein homeostasis. Furthermore, increased proteasome activity may prevent the accumulation of a critical mass of insoluble protein aggregates that can also inhibit the functioning of the proteasome.

### A greater proportion of immunoproteasomes may contribute to higher proteasome activity observed in naked mole-rats when compared to mice

Previous studies report augmented proteasome subunit expression when oxidative stress levels are increased [28], [29]. This is attributed at least in part to the oxidative stress induced stimulation of the (Nrf2) signaling pathway. Sulforaphane, a chemical inducer of Nrf2 signaling, triggered an increase in the gene expression and protein levels of the catalytic subunits β1, β2, and β5 and also, led to greater protection of murine neuroblastoma cells from H_2_0_2_-driven oxidative damage in a manner dependent on proteasomal function [29]. Interestingly constitutive Nrf2 protein levels in tissue samples from experimentally non-stressed naked mole-rats are three- to ten-fold higher than observed in samples from physiologically age-matched mice [52] and these high levels may be instrumental in maintaining proteasome content.

While 19S subunits show higher protein expression in naked mole-rats than in mice, the ‘housekeeping’ catalytic subunits (β2, β5) did not correspondingly increase. Rather, naked mole-rats have higher levels of immunoproteasome subunits (Figure 4 A, B). Immunoproteasomes reportedly are more efficient in degrading damaged proteins and have higher activity per proteasome unit than do housekeeping proteasomes [53]. Our biochemical data support this observation (Figure 1, 5) as immunoproteasomes also have elevated trypsin-like activity and a depressed PGPH activity [54]. Since trypsin-like activity shows the greatest interspecies differences (Figure 1, 5), it is likely, that these high levels in the naked mole-rat may be due to the formation of a sub-population of immunoproteasomes activated by heightened chronic oxidative stress.

Formation of immunoproteasomes reportedly is primarily triggered by a NFκB-mediated immune response or injury [32] and expression of immunoproteasome subunits is heightened by interferons or TNFα [53], [55]. There is, however, increasing evidence of a heterogeneous population of proteasomes in healthy liver tissue [56] and non-immunogenic immunoproteasome functions in that immunoproteasomes also degrade both oxidatively damaged nascent proteins and cytosolic proteins under conditions of oxidative stress [28], [53]. Furthermore, when the subunit (β5i) of the immunoproteasome is knocked out and the cells subjected to exogenous oxidative stress, the resultant impaired immunoproteasome function caused the formation of aggresomes [37], [53]. These studies confirm the importance of the β5i catalytic subunit in cleaving oxidatively damaged proteins and the role of the immunoproteasome in protein homeostasis.

Both NFκB and TNFα protein expression are higher in liver lysates of naked mole-rats when compared to mice (Figure 7). High levels of NFκB correlate with levels of the gene encoding the protein, inhibitor of nuclear factor kappa-B kinase subunit beta (IKK-β) the expression of which is four times higher in naked mole-rat liver tissue than in mice [6]. IKK-β through phosphorylation and subsequent UPS-mediated degradation of the inhibitor of NFκB (IκB), indirectly activates NFκB [57].

Nrf2 and NFκB are both also upregulated in response to oxidative stress [58], [59]. The high levels of oxidative damage in tissues from young, captive naked mole-rats [7] may reflect their perceived “hyperoxic response” to the gaseous atmosphere above ground. Naked mole-rats, having led a strictly chthonic existence since the early Miocene, have evolved to tolerate the hypoxic and hypercapnic conditions commonly found in subterranean habitats [1]. They naturally live in large social groups in plugged underground deep burrow systems. Here gas exchange is impeded and dependent upon soil porosity and the limited available oxygen for respiration is shared not only with conspecifics resting together, but also microbial organisms and plant roots [60]. This captivity-induced oxidative stress could also explain the observed high levels of Nrf2 [52], NFκB signaling (Figure 7) and downstream proteasome activation.

Given the fact that Nrf2 predominantly regulates ‘housekeeping’ proteasomes whereas NFκB regulates the immunoproteasome we question whether naked mole-rat non-immunogenic tissues contained hybrid proteasomes. The observed increase in proteasome activation in this study is partially regulated by standard UPS assemblies as well as immunoproteasome-related subunits since we see a concomitant increase in 19S and 11S subunits (Figure 4). These types of hybrid proteasomes also appear after interferon induction in HeLa cells, and constitutively in rat liver (low, less than 20%) and spleen (high, greater than 50%) [34]. In naked mole-rats these hybrid proteasomes would allow both a rapid response to oxidative stress in a non-ubiquitin dependent manner (11S cap/20S proteasome), and also would enable phenotypic responses to the changing microenvironment by upregulation of critical signaling pathways associated with cytoprotection (Nrf2) and the inflammatory response (NFκB) which is dependent on ubiquitin (19S cap/26S proteasome).

### In young animals, the cytosol is the site of proteasome-dependent cytoprotection

Proteasomes in the cytosolic fraction are primarily responsible for degrading regulatory proteins and those longer-lived proteins damaged over time, particularly in response to cellular stressors [12]. Not surprisingly, therefore, protein degradation in the naked mole-rat predominantly occurs in this fraction and both ChT-L and T-L activities in naked mole-rat cytosolic fractions are markedly higher than in those of mice (Figure 5, 6). There was no significant interspecific difference in activities in the microsomal fractions possibly indicating that the degree of mistranslation of the endoplasmic reticulum proteins or nascent protein misfolding is similar in both young mice and mole-rats. Nuclear proteasome activity was higher at two active sites in mice (ChT-L and PGPH; Figure 5) than in naked mole-rats suggesting that mice have higher turnover of transcription factors, and proteins involved in DNA-repair and DNA check point control [11], [17], [18]. It is unlikely that this reflects better DNA maintenance and repair in mice, although dysfunction in these processes can lead to increased mutations and cancer. Most strains of laboratory mice have a notoriously high predisposition to cancers [4] whereas naked mole-rats exhibit pronounced resistance to cancer [60]. Not only have we never observed incidences of spontaneous cancer in our captive 30 year maintained population [1] but naked mole-rat cells are also resistant to oncogenic transformation by Ras and sv40T antigen, known to induce aggressive invasive and metastatic tumors in rodents, bovines and primates [61]. Naked mole-rats also show significantly higher expression of several families of DNA repair genes than observed in mice [6]. It is possible that lower proteasome activity in the nuclear fraction leads to an extended half- life, and steady-state levels of these repair proteins thereby providing better maintenance of genomic integrity.

### In summary

We report that naked mole-rats show high levels of proteasome activity particularly in the cytosolic subcellular fraction where T-L activity is six-fold higher than that observed in mice. These high levels may reflect greater protein turnover, possibly in response to the chronic high levels of oxidative stress observed in naked mole-rats. Since both cytoprotective Nrf2 signaling and the NFκB- regulated inflammatory/immune response are triggered by oxidative stress [53], it is not surprising that the expression of these two critical transcription factors is significantly greater in naked mole-rats than in mice. These two signaling pathways are known to be involved in regulating proteasome activity and are likely modulators of naked mole-rat proteasome content and activity. Elevated signaling by both Nrf2 and NFκB may lead to the formation of not only a higher intracellular proteasome content but also more hybrid/immunoproteasomes even in liver tissues. Taken together, these data support the premise that regulated proteasome degradation pathways have an integral role in protein homeostasis by both degrading toxic proteins and modulating half-lives of key transcription factors. Future studies will determine whether naked mole-rats, during their prolonged apparent healthy aging, maintain efficacious high levels of removal of damaged proteins and proteostasis and whether or not this is a critical component of their attenuation of age-related decline and extended healthspan relative to that observed in short-lived mice.

## Methods

### Animals

All procedures involving animals were approved by the Institutional Animal Care and Use Committee at the University of Texas Health Science Center (San Antonio, TX) using protocol 11033-07-01-A. This study used two similar sized physiologically age-matched females from the rodent species C57BL/6 mice (4–6 months) and naked mole-rats (2–3 years). The mice were fed *ad libitum* a standard NIH-31 chow and were maintained in cohorts of four animals in microisolator mouse cages at 25°C, on a 12-h dark/light cycle. Naked mole-rats were from the well-characterized colonies of Dr. Rochelle Buffenstein housed at the University of Texas Health Science Center, San Antonio. Naked mole-rats were housed in simulated, multi-chambered burrow systems under constant climatic conditions that aimed to approximate their native habitat (30°C; 50% RH). Naked mole-rats were given an ad lib supply of fruit and vegetables supplemented weekly with a high protein and vitamin enriched cereal (Pronutro, South Africa). Animals were anesthetized with isoflourane, killed by cardiac exsanguination and the liver tissue immediately excised and flash frozen in liquid nitrogen.

### Whole Tissue Lysates and Subcellular Fractionation

Mouse and naked mole-rat liver lysates were separated into cytosolic, microsomal, and nuclear fractions using a modified Millipore Corp. procedure (2005) as previously described [16]. Briefly, the liver from a single animal was weighed and disrupted in a 2 mL Potter-Elvehjem homogenizer in RSB buffer (10 mM HEPES, pH 6.2, 10 mM NaCl, 1.4 mM MgCl_2_) at a weight-to-volume ratio of 1 g of tissue to 1 mL of buffer. The RSB buffer was supplemented with the addition of 1 mM ATP, 0.5 mM DTT, 5 mM MgCl_2_ to help maintain intact 26S subassemblies [62]. After twenty strokes, an aliquot of the homogenized liver was set aside for whole tissue lysate analysis. The rest of the homogenate was centrifuged at 2500×g for 6 min at 4°C. The supernatant was collected and the pellet was re-homogenized and centrifuged again under the same conditions two more times. The resulting supernatants were pooled and centrifuged at 13,000×g for 90 min (Ti70, Beckman Coulter, Fullerton, CA, USA). The collected supernatant from the high-speed centrifugation was labeled as the *cytosolic* fraction [16]. The final pellet from the initial homogenization step was washed again in RSB buffer, re-dissolved in RIPA buffer (10 mM Tris, pH 7.4, 10 mM NaCl, 5 mM MgCl2, and 1 mM DTT also supplemented with 1 mM ATP and 5 mM MgCl_2_) and then mixed for 2 h at 4°C on a lab rotator. Next, the material was centrifuged at 16,000×g for 10 min and the supernatant was designated as the *nuclear* fraction [16]. The pellet obtained in the ultracentrifugation step was re-suspended in RIPA buffer, mixed for 2 h on a lab rotator at 4°C followed by centrifugation at 10,000×g for 10 min. The resulting supernatant was labeled as the *microsomal* fraction [16]. Protein concentration was measured in all the fractions with the BCA Protein Assay (Pierce, Thermo Scientific, Rockford, IL, USA). The collected fractions were aliquoted into smaller volumes and then stored at −80°C until needed.

### Peptidolytic Activity Assay

The total peptidolytic activity of all three types of the proteasomal active sites was determined using fluorogenic model peptide substrates (obtained from Boston Biochem (Boston, MA) specific for each of the three classes of active centers: chymotrypsin-like (ChT-L), cleavage after hydrophobic residues (succinyl-LeuLeuValTyr-7-amido-4-methylcoumarin (Suc-LLVY-AMC)); trypsin-like (T-L), cleavage after basic residues (butoxycarbonyl-LeuArgArg-AMC (Boc-LRR-AMC)), and post-glutamyl peptide hydrolyzing activity (PGPH), post-acidic residue cleavage, (carbobenzoxy-LeuLeuGlu-AMC (Z-LLE-AMC)) as previously described [16], [40], [41]. Parallel assays were run with varying concentrations of N-(benzyloxycarbonyl) leucinyl-leucinylleucinal (MG132) proteasome inhibitor (Calbiochem, San Diego, CA) ranging from 10 µM to 250 µM to determine non-proteasomal AMC release. We discovered during the course of this titration that 20 µM (microsomal and nuclear), 50 µM (whole lysates) and 150 µM (cytosolic) ablated proteasomal activity in naked mole-rat preparations until a plateau of effect was achieved where an increase in MG132 concentration did not further inhibit proteasome activity [63]. As reported previously, mouse samples were inhibited in that manner at concentrations of 10 to 20 µM of MG132 [16], [39]. This non-specific activity was subtracted from the rate measured in the absence of the inhibitor. To confirm the specificity of the MG132 proteasome inhibitor [32], [39], activity was also measured in the absence and presence of adamantane-acetyl-(6-aminohexanoyl)_3_-(leucinyl)_3_-vinyl-(methyl)-sulfone (Ada-(Ahx)_3_-(Leu)_3_-VS; Calbiochem), another well characterized proteasome-specific inhibitor [16]. The pattern of Ada-(Ahx)_3_-(Leu)_3_-VS inhibition induced the same level of inhibition to that observed with MG132 in our sample preparations. Specific peptidolytic activity of proteasome was presented as pmol of released AMC in 1 min per 1 µg of total protein in the test sample. This was determined after generating a standard curve using serial dilutions of 1 µM AMC (Calbiochem, San Diego, CA) and measuring the fluorescence using a SpectraMax Multi-mode microplate reader (excitation 355 nm, emission 460 nm) (Molecular Devices, Sunnyvale, CA).

### Native Gel Electrophoresis

Native gel electrophoresis has been used to determine if the proteasome remains intact in a higher molecular weight form (i.e. 26S) or exists disassembled during the assay (20S) [64]. This could reflect the physiological properties of the tissue tested or whether or not the extraction process leads to disassembly. Fifty micrograms of fractionated lysate from each of the sample groups prepared as described in Subcellular Fractionation above (q.v) were run on a 3–12% non-denaturing, gradient polyacrylamide gel (Invitrogen, Carlsbad, CA). The gel was run at 30 V for 30 min in a 4°C cold cabinet, thereafter the voltage was increased to 35 V for 1 hour, 50 V for 1 hr and further increased to 75 V for three more hours [43], [64]

Peptidolytic activity of proteasomes was detected after incubating the gels in a Suc-LLVY-MCA substrate dissolved in 50 mM Tris pH 8.0, 5 mM MgCl_2_, 1 mM DTT, 1 mM ATP, and 0.02% SDS for 15, 30 and 60 min at 37°C. Proteasome bands were identified by the release of highly fluorescent, free AMC [64], [65]. Following the in-gel assay, the protein from the gel was transferred to PVDF via a wet transfer procedure (see below) and subjected to Western blotting analyses to identify the various proteasome subunits and whether the proteasome remained intact or was disassembled into 20S, 19S, or other complexes.

### Western Blot Analysis of Proteasome Subunits

A wet transfer method was used to electrophoretically transfer proteins from the native gel to a PVDF. These membranes were then probed with antibodies specifically recognizing the α7 and RPT5 subunits (Enzo Life Sciences, Plymouth Meeting, PA, USA). In addition liver tissue lysates were fractionated in 12% SDS-PAGE (Biorad Life Sciences, Hercules, CA) and transferred to PVDF membranes (Biorad Life Sciences, Hercules, CA). The membranes were probed with antibodies against the following proteasome subunits: α4 (mouse mAb, 1∶1000, PW8120), α7 (mouse mAb, 1∶1000, PW8110), β2 (mouse mAb, 1∶1000, PW9300), MECL1 (β2i) (rabbit pAb, 1∶1000, PW8350), LMP7 (β5i) (mouse mAb, 1∶1000, PW8845), β4 (rabbit pAb, 1∶1000, PW8890), Rpn7(rabbit pAb, 1∶1000, PW8225), Rpn10 (mouse mAb, 1∶1000, PW9250), Rpt5 (mouse mAb, 1∶1000, PW8770) (Enzo Life Sciences, Plymouth Meeting, PA, USA), and PA28α (goat pAb, 1∶500, sc-21267) (Santa Cruz Biotechnology, Santa Cruz, CA, USA). Antibodies against NFκB (rabbit pAb, 1∶1000, ab16502) (abcam, Cambridge, MA, USA) and TNFα (rabbit, pAb, 1∶1000, NB600-587) (Novus Biologicals, Littleton, CO, USA) were also used. Actin (mouse mAb, 1∶5000, CP-01) was used as a loading control (Calbiochem/EMD Biosciences, Rockland, MA). HRP-conjugated secondary antibodies for rabbit (1∶5000), mouse (1∶5000), or goat (1∶10,000) (Santa Cruz Biotechnology, Santa Cruz, CA) were used to visualize the immune-reaction using the ECL Prime Western Blotting Detection Reagent, a chemiluminescent substrate (Amersham, Buckinghamshire, UK). Immunoblots were quantified using the Typhoon 9410 variable mode imager (GE Healthcare) and the ImageJ public domain Java image processing program (http://rsbweb.nih.gov/ij/).

### Statistical Analysis

Livers from twelve naked mole-rats and twelve mice were analyzed. A two-tailed Student's T-test on two different statistical platforms (Microsoft Excel 2010; SigmaPlot v. 11) was used to determine significant differences in the means for the peptidolytic assays. One-way ANOVA was used in the subcellular fraction experiments to analyze the variances between fractions and species (SigmaPlot v. 11) Statistical significance was set at the p<0.05 level.
